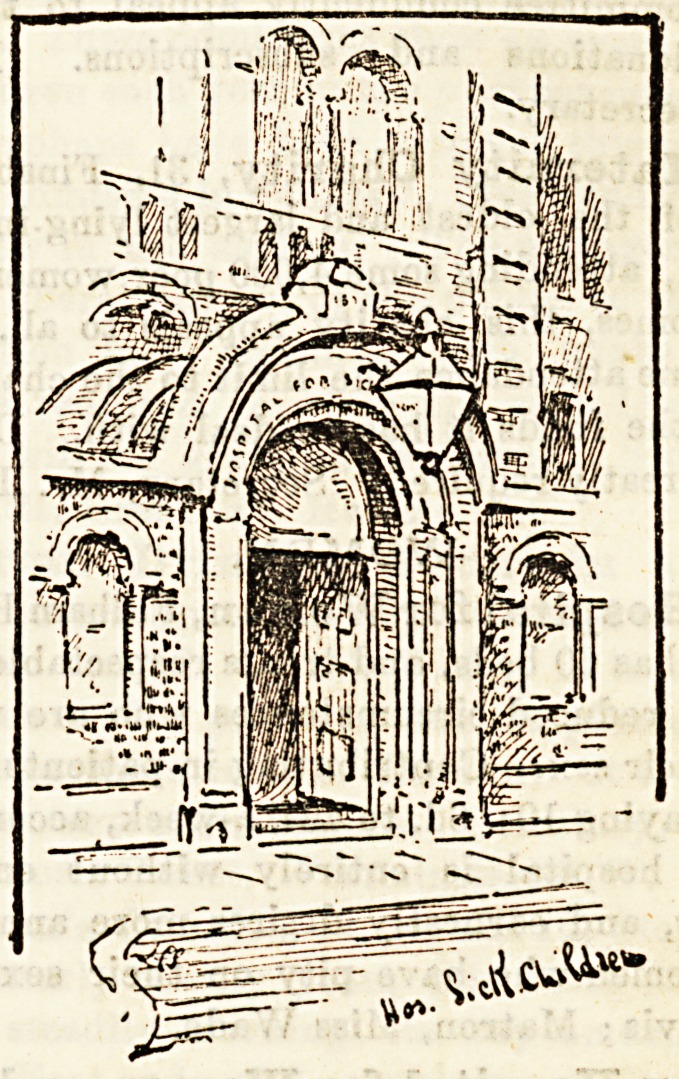# Children

**Published:** 1892-12-24

**Authors:** 


### CHILDREN.

**East London Hospital for Children,** Shadwell, E.—An excellent institution, the original home of which in Ratcliff Highway was graphically described by Charles Dickens, whose memorable account will live for generations. Unfortunately, the children admitted are in as dire straits now as then, and funds are sadly needed. Secretary, Mr. S. Whitford; Matron, Mrs. Fisher; Lady Superintendent, Miss F. A. Davies.

**Evelina,** Southwark Bridge Road, S.E.—The only hospital in South London exclusively for children, and situated in a district where nearly all the resident inhabitants may be numbered among the poor. Any poor sick or suffering child is admitted if there is room. It is open to visitors every day, rom two to four. Contributions, which could not be better bestowed, may be sent to the Secretary, Mr. T. S. Chapman; Lady Superintendent, Miss Alice Cross.

**Hospital for Sick Children,** Great Ormond Street, W.C.—This was the first hospital in the kingdom specially designed for, and devoted to the reception of sick children. The 127 beds which the hospital at present contains being found totally inadequate, a new wing has been erected with 93 additional cots. This is now completed, but a further £8,000 must be collected to pay for furniture and fittings. Besides this, money is still required to meet the expenses for the last quarter of this year. Seeing that this is the largest children's hospital in Great Britain, that it is excellently managed, and that it has treated over 1,400 in-patients and 20,600 out-patients during 1891, we do not think that this year's appeal to the charitable public for funds to enable them to carry on the great work will be made in vain. Secretary, Mr. Adrian Hope; Lady Superintendent, Miss K. Hendle Close.

**North-Eastern Hospital for Children,** Hackney Road, E.C.—This institution has prospered greatly since Mr. Alfred Nixon became Secretary some years ago, and its present condition shows what can be done by an energetic management. The average number of out-patients during the last ten years is 13,500, and last year 702 in-patients were treated. Patients are admitted from all parts of London and the United Kingdom. Secretary, Mr. Alfred Nixon; Matron, Miss E. W. Curno.

**Paddington Green Children's Hospital,** W.—Though this hospital is in the fortunate position of being free from debt on the current expenditure, yet it this year makes a special appeal for the purpose of raising funds to enable the committee to add a wing in order to provide as far as possible, for the number of cases which they are now obliged to reject for want of room. The cost will probably amount to £4,000 or £5,000, but the committee have determined not to commence building until a considerable proportion of this has been raised. Secretary, Mr. W. H. Pearce; Matron, Miss Anderson.

[illegible]r [illegible] **for Children,** Queen's Road, Ch[illegible]lsea.—Established at Chelsea in 1866 for the relief of the sick and suffering children of the poor, this unendowed hospital seeks further subscriptions to help it to carry on its work. The expenditure, which is only 19s. per patient, yet reaches £5,000 to £6,000 a-year, and this has to be raised entirely by voluntary subscriptions. Secretary, Commander W. C. Blount, R.N.; Matron, Miss Cooper.

## Figures and Tables

**Figure f1:**